# Early Mortality in Patients who Received Extensive Surgical Management for Acute Type A Aortic Dissection - Analysis of 452 Consecutive Cases from a Single-center Experience

**DOI:** 10.21470/1678-9741-2019-0258

**Published:** 2020

**Authors:** Ahmed Sayed Abdelhameed, Feng Xin, Xiang Wei

**Affiliations:** 1 Department of Cardiothoracic Surgery, Huazhong University of Science and Technology, Wuhan, Hubei, People’s Republic of China.

**Keywords:** Coronary Artery Disease, Risk Factors, Multiple Organ Failure, Aneurysm, Dissecting, Shock, Heart Failure, Logistic Models, Stroke

## Abstract

**Objective:**

To detect the potential risk factors associated with early mortality in patients who received extensive surgical management, in the form of total arch replacement plus frozen elephant trunk and arch debranching (hybrid repair technique), for acute type A aortic dissection.

**Methods:**

The clinical and surgical data of 452 surgically treated patients with acute type A aortic dissection at our center, between March 2010 and December 2016, have been retrieved. Uni and multivariate logistic regression analyses were carried out to detect the effect of various preoperative demographics and different perioperative variables on early mortality.

**Results:**

Overall 30-day mortality occurred in 70 out of 452 patients (15.4%). The principal causes of death were multiple organ failure (n=38), cardiac failure (n=18), and severe pulmonary infection (n=10). Risk factors for early mortality were identified with multivariate analysis. Preoperatively, overweight (*P*<0.025), alcohol drinking (*P*<0.002), coronary artery disease (*P*<0.014), hemodynamic shock (*P*<0.006), and elevated white blood cells count (*P*<0.002) were associated with higher mortality rate. Postoperatively, prolonged operation time (*P*<0.008), stroke (*P*<0.0001), and acute renal dysfunction (*P*<0.0001) were highly associated with death.

**Conclusion:**

Considering the advantages of extensive surgical management for acute type A aortic dissection over the other less aggressive surgical approaches, it should be advised whenever indicated, provided that being carried out by experts in the field of adult aortic surgery in high-volume centers. The surgeon should be aware of the patient’s preoperative comorbidities and other risk factors for early mortality, in particular, prolonged operation time.

**Table:** 

Abbreviations, acronyms & symbols				
AAA	= Abdominal aortic aneurysm		ICU	= Intensive care unit
AAAD	= Acute type A aortic dissection		IQR	= Interquartile range
AD	= Aortic dissection		IRAD	= International Registry of Acute Aortic Dissection
ARD	= Acute renal dysfunction		LVEF	= Left ventricular ejection fraction
AUC	= Area under the curve		MC	= Mainland of China
BMI	= Body mass index		ROC	= Receiver operating characteristics
CA	= Circulatory arrest		RTAD	= Retrograde type A aortic dissection
CABG	= Coronary artery bypass grafting		SD	= Standard deviation
CAD	= Coronary artery disease		SPSS	= Statistical Package for the Social Sciences
COPD	= Chronic obstructive pulmonary disease		TAR	= Total aortic arch replacement
CPB	= Cardiopulmonary bypass		TEVAR	= Thoracic endovascular aortic repair
DSWI	= Deep sternal wound infection		TIA	= Transient ischemic attack
DTA	= Descending thoracic aorta		TND	= Transient neurologic dysfunction
ESM	= Extensive surgical management		WBCs	= White blood cells
FET	= Frozen elephant trunk ok		WHO	= World Health Organization
GERAADA	= German Registry of Acute Aortic Dissection Type A			

## INTRODUCTION

In acute type A aortic dissection (AAAD), the complications of either aortic rupture or malperfusion syndromes may arise if the remaining distal portion of the aorta is untreated^[1]^. As a result, recent surgical practice advocates a more aggressive repair approach using total aortic arch replacement (TAR) combined with the frozen elephant trunk (FET) technique^[2,3]^. Arch debranching is a safe alternative to FET in AAAD patients and it has the advantage of being performed without circulatory arrest in addition to being suitable in candidates who are unfit for FET^[4-6]^.

Over the past few decades, risk factors related to mortality of AAAD have been explored widely but with a relatively small number of patients who received extensive surgical management (ESM) for AAAD. Contrarily, our study is one of the few studies in the literature which investigated a relatively large series of patients who underwent ESM for AAAD. The dominant impact factors on adverse surgical outcomes of AAAD stay a matter of controversy. This might be explained by the rarity of the disease and the lack of numerous experienced surgical centers, which resulted in lack of clear risk stratification. Also, the emergency nature and the evolving surgical techniques necessitated continuous update regarding any recognizable predictors that could aid aortic surgeons in their daily practice. The aim of the present study is to explore the effect of different peri and intraoperative variables on early mortality in a large series of patients who underwent extensive surgical repair for AAAD.

## METHODS

This study was approved by the institutional ethical review board and informed consent was waived as this is a retrospective study of pre-existing data. Clinical and surgical data of AAAD patients who have been treated at our aortic surgery center between March 2010 and December 2016 were collected from the institutional database. Four independent aortic surgeons performed the surgical intervention.

At our institution, the FET technique was the primary management strategy in 364 out of 452 patients (80.5%), being indicated to patients who required an extensive repair for aortopathy. Eighty-eight out of 452 patients (19.5%) underwent arch debranching (hybrid repair) for AAAD.

Diagnosis of AAAD was confirmed by multidetector computed tomographic angiography. AAAD was defined as acute if chest pain or other related symptoms were present for less than two weeks before presentation to our hospital.

Extensive repair was indicated when the site of the intimal tear was in the aortic arch, close to the descending thoracic aorta (DTA), or when the supra-arch branches were involved by the dissection process, as well as to patients with Marfan syndrome or in cases of dissection with a dilatated aortic arch.

High-risk status, obesity, surgeon’s preference, and patient’s willingness were our indications for hybrid repair, provided that the anatomy was suitable for this kind of intervention.

Excluded cases were those with chronic aortic dissection (AD) (54 cases), patients who did not undergo surgical treatment (22 cases), and those who had undergone open surgery for retrograde type I AD post thoracic endovascular aortic repair (TEVAR).

**Fig. 1:**
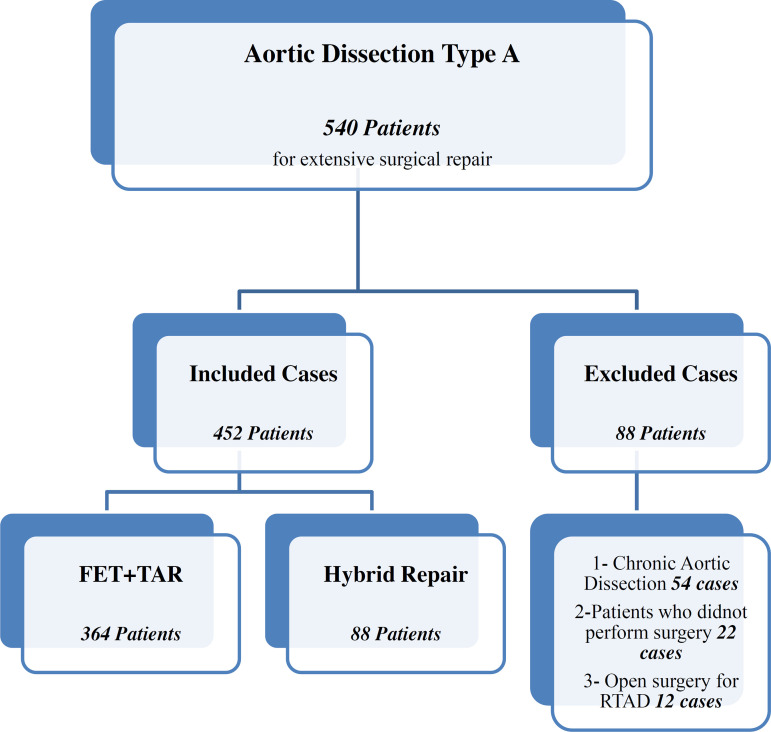
Flow chart picture showing included and excluded cases. FET=frozen elephant trunk; RTAD=retrograde type A aortic dissection; TAR=total aortic arch replacement

The flow chart picture (Figure 1) shows included and excluded cases.

Hemodynamic shock was defined as a drop in systolic blood pressure below 90 mmHg at the emergency department. Emergency surgery was the operation carried out within 24 hours after admission. Postoperative acute renal dysfunction (ARD) was defined as a serum creatinine level three-fold greater than baseline (≥ 4 mg/dl), urine output < 0.3 ml/kg/h for 24 hours, anuria for 12 hours, or a new requirement for transient hemodialysis.

Multiple organ dysfunction syndrome was defined as existence of at least two or more of the following conditions: acute liver failure, acute respiratory distress syndrome, acute heart failure, acute renal failure, diffuse or focal neurologic ischemic damage, such as permanent paraparesis or paraplegia due to deterioration of blood supply to the spinal cord or signs of central neurological damage following cerebral hypoperfusion, and septicemia.

### Statistical Analysis

The Statistical Package for the Social Sciences (SPSS) software (IBM, Armonk, NY, USA), version 22.0, was used for analysis. Continuous variables were presented as mean ± standard deviation and median with interquartile range. Nominal variables were compared by c^[2]^ tests or two-sided Fisher’s exact tests. Continuous univariate predictors for death were tested using Student’s *t-*tests or Wilcoxon-Mann-Whitney tests, as appropriate. A *P*-value < 0.05 was considered statistically significant. In order to assess the predictive risk factors for mortality and morbidity, each variable that was considered significant at univariate analysis was selected for multivariable analysis at three levels: preoperative, intraoperative, and postoperative, independently.

Receiver operating characteristics (ROC) curves were performed to estimate the capacity of prolonged operation and cardiopulmonary bypass (CPB) time in predicting mortality.

### Operative Strategies and Techniques

**Fig. 2:**
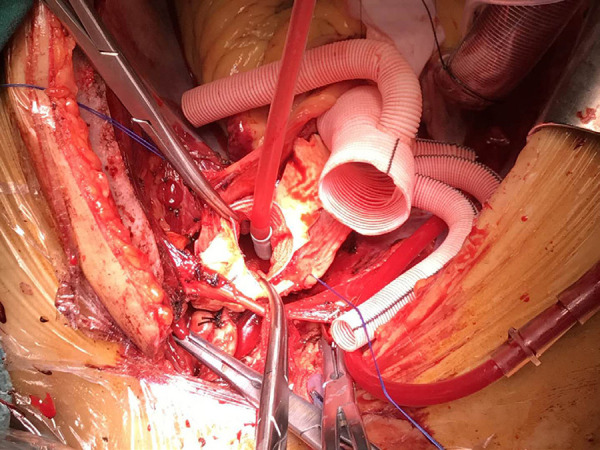
Implanting the stented graft into the descending aorta under direct vision, followed by total arch replacement with a fourbranched vascular graft (Hemashield Platinum; Maquet, Wayne, NJ, USA); a specific anastomotic order for aortic reconstruction is conducted starting by the proximal descending aorta, followed by the left carotid artery, then the ascending aorta, the left subclavian artery, and lastly, the innominate artery.

**Fig. 3:**
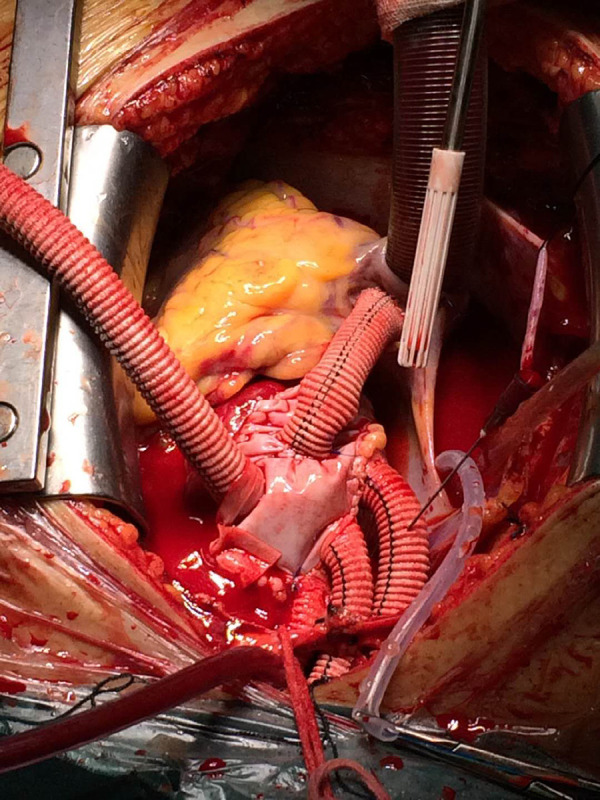
Final reconstruction with frozen elephant trunk+total aortic arch replacement.

For FET technique, general anaesthesia, supine position, and sterile draping were performed followed by exposure of the right axilla at the beginning. Then a median sternotomy was done and CPB establishment was made by placing an arterial cannula in the right axillary artery and a two-stage cannula for venous drainage. Circulatory arrest was started after completing the proximal aortic repair at 20-28 ˚C nasopharyngeal temperature. Our cerebral perfusion strategy was to use the right axillary artery alone for antegrade cerebral perfusion at a rate of 6±2 ml/kg/min. If there was a poor backward bleeding from the left-sided arch vessels, then bilateral antegrade cerebral perfusion was preferred by placing an additional balloon-tipped catheter into the lumen of the left carotid artery. We have used the Cronus prosthesis (Microport Medical, Shanghai, China), which is commercially available in China, to perform the Sun’s procedure^[7]^, that involves implanting the stented graft into the descending aorta under direct vision, followed by total arch replacement with a four-branched vascular graft (Hemashield Platinum, Maquet, Wayne, NJ, USA). A specific anastomotic order for aortic reconstruction was conducted starting by the proximal descending aorta, followed by the left carotid artery, then the ascending aorta, the left subclavian artery, and lastly, the innominate artery (Figure 2). Rewarming and reperfusion were initiated just after the distal anastomosis to minimize cerebral and coronary ischemia (Figure 3).

As regards to the arch debranching procedure, the same abovementioned steps were carried out plus placing an additional arterial cannula in the femoral artery to establish CPB. Use of the four-branch graft to replace the ascending aorta, starting by proximal anastomosis first, then construction of the distal anastomosis beyond the level of the innominate artery, was done. Afterward, we removed the aortic clamp and started rewarming. The arch vessels were anastomosed to the limbs of the artificial graft on pump after heavy ligatures of each branch vessel were taken off. If there was a difficult left subclavian artery, we rerouted it to the left axillary artery. After weaning off CPB, administration of a half dose of protamine was done for heparin neutralization.

Placing the endovascular stent was done before sternal closure, using the femoral artery for the stent delivery. In order to achieve about 20% oversize in diameter of the stent graft (range 28-34 mm), we were guided by the size of the pre-sutured four-branch Dacron graft. Retrograde advancement of the stiff guidewire was done from the femoral artery up to the left ventricle or by using the fourth limb of the Dacron. The implementation of the stent graft in the Dacron graft was done with an overlapping margin of about 2 cm. Occasionally, in order to cover the DTA, we needed two stent grafts instead of one, as one stent graft (15-17 cm in length) might be insufficient to cover DTA. There was no need to extend beyond the level of T8 vertebra in most cases to cover the distal territory. Assessment of endoleak or incomplete sealing was made by repeating the angiography^[4]^.

## RESULTS

Four hundred fifty-two surgically treated AAAD patients were included in this study. Of these, 70 patients (15.4%) died within 30 days. Regarding hybrid repair, 13 patients died out of 88 cases (14.7%), while 57 patients died out of 364 cases (15.6%) when FET+TAR technique was used. The principal causes of death were multiple organ failure (n=38), cardiac failure (n=18), and severe pulmonary infection (n=10). Two deaths were due to deep sternal infection and two deaths occurred due to surgical failure. Detailed information on demographics and peri and intraoperative data are presented in Tables 1 to 6.

**Table 1:** Patients' demographics data.

Variable	Value (%)
Age (years)	
Mean ± SD	48.36±9.57
Median (IQR)	48 (42-53)
Gender	
Male (%)	346 (76.2)
Weight (kg)	
Mean ± SD	72.05±12.44
Median (IQR)	71 (64-80)
Smoking (%)	200 (44.1)
Alcohol drinking (%)	172 (37.9)
Previous cardiac surgery (%)	12 (2.6)
Duration of complaint	
0-24 hours (%)	254 (55.9)
> 24 hours (%)	188 (41.4)
Diabetes mellitus	44 (9.7)
Hypertension (%)	356 (78.4)
Hyperlipidemia (%)	30 (6.6)
COPD (%)	14 (3.1)
CAD (%)	16 (3.5)
Aortic regurgitation ≥ grade 3	84 (18.5)
Shock (%)	14 (3.1)
Malperfusion (%)	
Stroke (%)	6 (1.3)
TIA (%)	2 (0.4)
Myocardial ischemia (%)	116 (25.6)
Renal dysfunction (%)	36 (7.9)
Mesenteric ischemia (%)	0
Lower extremity (%)	2 (0.4)
Marfan syndrome (%)	18 (3.9)
LVEF%	
Mean ± SD	60.5±6.1
Median (IQR)	60 (58-63.2)
Elevated WBCs count (%)	246 (54.2)
Anemia (%)	84 (18.5)
Low platelets count (%)	174 (38.1)

Values are presented as mean and SD and median with IQR or n (%). CAD=coronary artery disease; COPD=chronic obstructive pulmonary disease; IQR=interquartile range; LVEF=left ventricular ejection fraction; SD=standard deviation; TIA=transient ischemic attack; WBCs=white blood cells

**Table 6:** Early outcomes (univariate and multivariate analyses).

Variable	Univariate *P*-value	Multivariate (*P*-value)
30-day mortality (%)		
Stroke (%)	< 0.0001	< 0.0001
TND (%)	0.997	
Hemiplegia (%)	0.053	
DSWI (%)	0.706	
ARD (%)	< 0.0001	< 0.0001
Pulmonary infection (%)	0.009	
Tracheotomy (%)	0.002	
Ventilation time (h)	0.001	
Mean ± SD		
Median (IQR)		
ICU readmission (%)	< 0.0001	

ARD=acute renal dysfunction; DSWI=deep sternal wound infection; ICU=intensive care unit; IQR=interquartile range; SD=standard deviation; TND=transient neurologic dysfunction

### Preoperative Risk Factors for Death

The risk of death was significantly increased on multivariable analysis in relation to body weight (*P*<0.025), when the patient was positive for alcohol drinking history (*P*<0.002) or shocked prior to surgery (*P*<0.006).

Furthermore, concomitant coronary artery disease (CAD) (*P*<0.014) and elevated leucocytic count were also associated with an increased risk of early death (*P*<0.002) on multivariate logistic regression analysis (Tables 1 and 2).

**Table 2:** Patients' demographics data (univariate and multivariate analyses).

Variable	Univariate *P*-value	Multivariate (*P*-value)
Age (years)	0.855	
Mean ± SD		
Median (IQR)		
Gender	0.174	
Male (%)		
Weight (kg)	0.063	0.025
Mean ± SD		
Median (IQR)		
Smoking (%)	0.869	
Alcohol drinking (%)	0.014	0.002
Previous cardiac surgery (%)	0.999	
Duration of complaint		
0-24 hours (%)	0.047	
> 24 hours (%)	0.428	
Diabetes mellitus	0.733	
Hypertension (%)	0.845	
Hyperlipidemia (%)	0.345	
COPD (%)	0.282	
CAD (%)	0.124	0.014
Aortic regurgitation ≥ grade 3	0.509	
Shock (%)	0.010	0.006
Malperfusion (%)		
Stroke (%)	0.999	
TIA (%)	>0.999	
Myocardial ischemia (%)	0.130	
Renal dysfunction (%)	0.054	
Mesenteric ischemia (%)		
Lower extremity (%)	>0.999	
Marfan syndrome (%)		
LVEF%		
Mean ± SD		
Median (IQR)		
Elevated WBCs count (%)	0.043	0.002
Anemia (%)	0.206	
Low platelets count (%)	0.768	

Values are presented as mean and SD and median with IQR or n (%). CAD=coronary artery disease; COPD=chronic obstructive pulmonary disease; IQR=interquartile range; LVEF=left ventricular ejection fraction; SD=standard deviation; TIA=transient ischemic attack; WBCs=white blood cells

### Intraoperative Risk Factors for Death

**Fig. 4:**
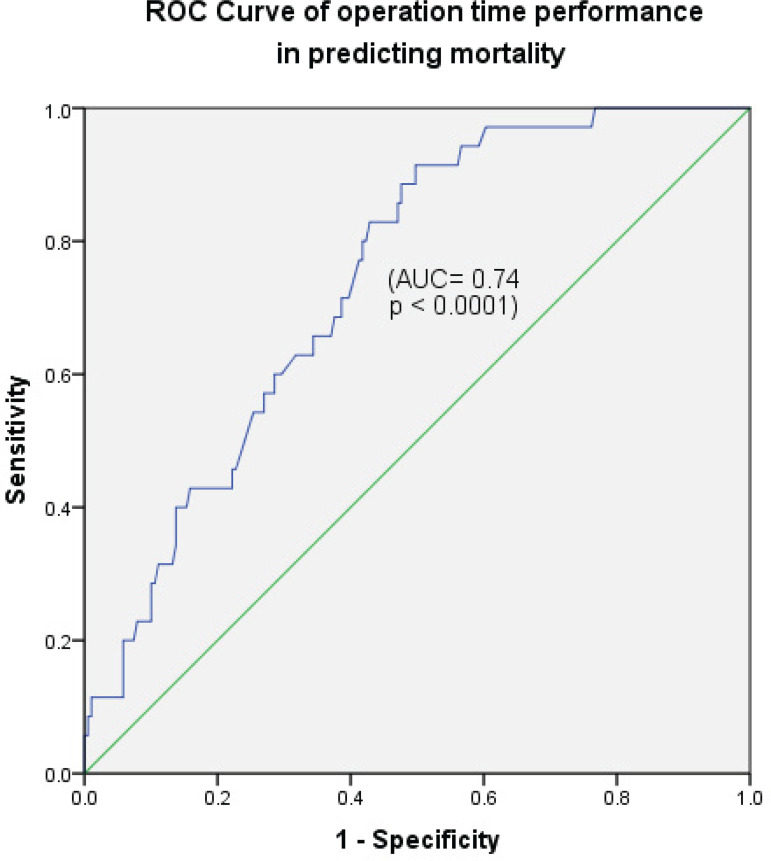
Cutoff point of 511.5 minutes (8.525 hours) had an accuracy of 74.1% in predicting mortality (sensitivity 73.3%, specificity 60%, P<0.0001). AUC=area under the curve; ROC=receiver operating characteristics

**Fig. 5:**
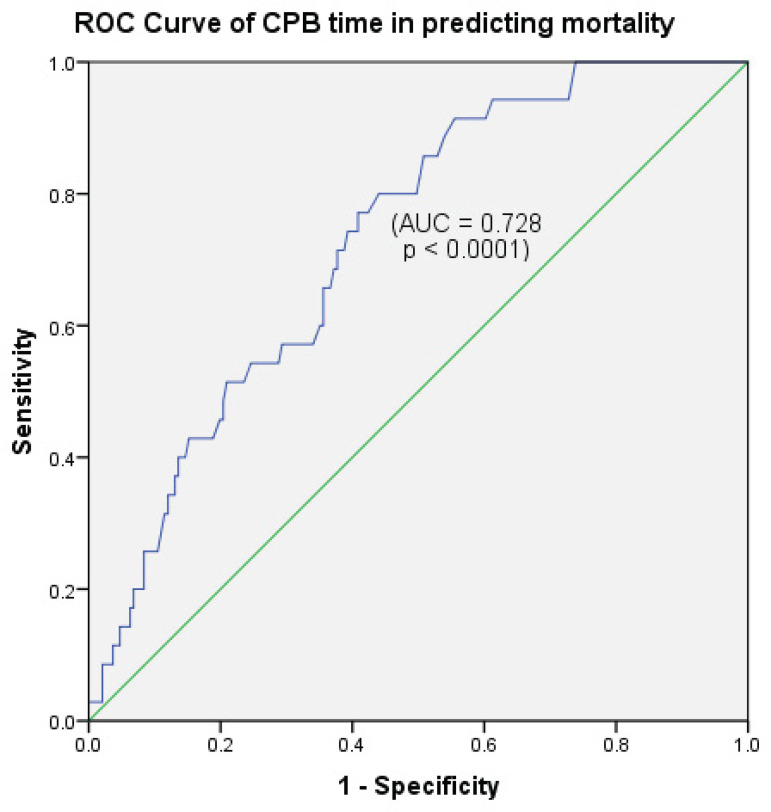
Cutoff point of 223 minutes (3.72 hours) had an accuracy of 72.8% in predicting mortality (sensitivity 73.5%, specificity 60.1%, P<0.0001). AUC=area under the curve; CPB=cardiopulmonary bypass; ROC=receiver operating characteristics

Prolonged operation and CPB times were highly significant predictors of early mortality on univariable logistic regression analysis (*P*<0.0001) and the entire surgery time remained significantly correlated with mortality in the multivariate analysis (*P*<0.008) (Tables 3 and 4). ROC curves of operation time and CPB time performance in predicting mortality showed area under the curve of 74.1% (*P*<0.0001) and 72.8% (*P*<0.0001), respectively. A selected cutoff point of 511.5 minutes (8.525 hours) of operation time had sensitivity of 73.3% and specificity of 60% (Figure 4), and a cutoff value of 223 minutes (3.72 hours) of CPB time showed sensitivity of 73.5% and specificity of 60.1% in predicting mortality (Figure 5).

**Table 3:** Operative variables.

Variable	Value (%)
Emergency (%)	212 (46.7)
Hybrid (%)	88 (19.5)
Bentall (%)	150 (33)
CABG (%)	54 (11.9)
Arterial cannulation method	
Right axillary artery	400 (88.1)
Right axillary & right femoral arteries	36 (7.9)
Distal ascending aorta	6 (1.3)
Left femoral artery	6 (1.3)
Operation time (min)	
Mean ± SD	513.55±101.27
Median (IQR)	501 (440-565)
Clamp time (min)	
Mean ± SD	120.88±31.57
Median (IQR)	122 (96-139)
CPB time (min)	
Mean ± SD	225.18±55.01
Median (IQR)	217 (189.5-251.25)
CA time (min)	
Mean ± SD	26.77±8.73
Median (IQR)	26 (21-30.75)
Nasopharyngeal temperature (˚C)	
Mean ± SD	21.46±4.16
Median (IQR)	
Cerebral perfusion	
Unilateral antegrade	328 (72.2)
Bilateral antegrade	92 (20.3)

CA=circulatory arrest; CABG=coronary artery bypass grafting; CPB=cardiopulmonary bypass; IQR=interquartile range; SD=standard deviation

**Table 4:** Operative variables (univariate and multivariate analyses).

Variable	Univariate *P*-value	Multivariate (*P*-value)
Emergency (%)	0.614	
Hybrid (%)	0.521	
Bentall (%)	0.909	
CABG (%)	0.299	
Arterial cannulation method	0.844	
Right axillary artery		
Right axillary & right femoral arteries		
Distal ascending aorta		
Left femoral artery		
Operation time (min)	< 0.0001	< 0.008
Mean ± SD		
Median (IQR)		
Clamp time (min)	0.014	
Mean ± SD		
Median (IQR)		
CPB time (min)	0.0001	
Mean ± SD		
Median (IQR)		
CA time (min)	0.422	
Mean ± SD		
Median (IQR)		
Nasopharyngeal temperature (˚C)	0.278	
Mean ± SD		
Median (IQR)		
Cerebral perfusion	0.239	
Unilateral antegrade		
Bilateral antegrade		

CA=circulatory arrest; CABG=coronary artery bypass grafting; CPB=cardiopulmonary bypass; IQR=interquartile range; SD=standard deviation

### Postoperative Risk Factors for Death

Both stroke and ARD were highly significant predictors of early mortality on multivariable analysis (*P*<0.0001) (Tables 5 and 6).

**Table 5:** Early outcomes.

Variable	Value (%)
30-day mortality (%)	70 (15.4)
Mortality in hybrid repair (%)	13 (14.7)
Mortality in FET+TAR (%)	57 (15.6)
Stroke (%)	30 (6.6)
TND (%)	168 (37)
Hemiplegia (%)	2 (0.4)
DSWI (%)	20 (4.4)
ARD (%)	68 (15)
Pulmonary infection (%)	144 (31.7)
Tracheotomy (%)	74 (16.3)
Ventilation time (h)	
Mean ± SD	124.25±122.94
Median (IQR)	89 (40-165)
ICU readmission (%)	50 (11)

ARD=acute renal dysfunction; DSWI=deep sternal wound infection; FET=frozen elephant trunk; ICU=intensive care unit; IQR=interquartile range; SD=standard deviation; TAR=total aortic arch replacement; TND=transient neurologic dysfunction

## DISCUSSION

Our surgical strategies - TAR+FET and hybrid repair - are aggressive repair techniques that showed better results for the management of AAAD^[8]^. Validation of the FET technique has been acquired as a compatible way for repair of AAAD^[9]^. In the meantime, various techniques for aortic debranching were designed to achieve AAAD repair with promising outcomes^[5,6]^.

In the year 2000, the International Registry of Acute Aortic Dissection (IRAD) group reported a 58% in-hospital death rate^[10]^ in patients who received medical treatment for AAAD. Even when surgery is conducted in a prompt time, the adverse outcome and death rates for AAAD are still high. Other large AAAD studies, *e.g.* the Chinese study (mainland of China [MC]; n=270, in 2011) or IRAD (n=617, in 2004), reported mortality rates of 37.0 and 30.6%^[11,12]^, respectively. Another important and relatively recent study is the German Registry of Acute Aortic Dissection Type A (GERAADA)^[13]^, which investigated the early mortality of 2,137 patients after surgical intervention for AAAD (16.9%), being this the biggest number of patients till now included in an AAAD early mortality study. GERAADA’s results should define the up-to-date degree of excellence and experience in surgical management of AAAD comparable to other studies, in which 17% is the average death rate. It is noteworthy to mention that these studies had a big number of patients, but a small percentage of ESM. The great decrease in death rate is attributed to the continuous surgical advance; not only improvements in surgical techniques, but also in temperature management and perfusion strategies, as well as anaesthesia refinements.

### Risk Factors for Mortality in Acute Type A Aortic Dissection Patients

The effect of obesity on perioperative and late postoperative outcomes in acute AD remains controversial. However, obese patients are significantly complex candidates to surgery in acute AD due to the demanding anatomy and difficulty of arterial access.

According to accessible data in Asia, a World Health Organization (WHO) expert consultation stated that the body fat content of Asians is generally higher than of white races of the same sex, age, and body mass index (BMI). Additionally, Asians are more vulnerable to develop type II diabetes mellitus and cardiovascular illness even if their BMI is lower than 25 kg/m^2^. Hence, the present WHO cutoff points for BMI are insufficient for assessment of overweight and obesity in Asian populations^[14]^.

In this study, increased body weight was an independent factor of early mortality. Being overweight was associated with early mortality as stated in a Chinese study by Ma et al.^[5]^. Interestingly, another Western study demonstrated that obesity (BMI > 30 kg/m^2^) is significantly related to early mortality in AAAD patients^[15]^.

In our study, a positive history of alcohol consumption was also significantly related to early mortality in univariate analysis and remained statistically significant in the multivariate logistic regression analysis. It has been stated that alcohol consumption in moderation might have a protective effect against CAD and ischemic stroke^[16]^, however, the relationship between alcohol consumption and aortic diseases are not so clear. Several observational studies reported either positive or inverse and even U-shaped relationship between alcohol intake and abdominal aortic aneurysm (AAA)^[17]^. A recent Japanese cohort study suggested the same protective effect of light to moderate alcohol consumption against mortality in aortic diseases^[17]^; this finding might be supported by the anti-atherogenic effect of alcohol^[18,19]^. Atherosclerosis is a cardinal pathological feature in AAA^[22]^ but not in thoracic aortic aneurysm or dissection^[20]^. On the other hand, elevated blood pressure is one of the effects of chronic alcohol consumption^[21]^, which is a major culpable factor in the development of AD. Our finding must be further investigated as regards to drinking status, habits, and amount, beverage type, and average amount on one occasion for regular drinkers, as little is known about the relationship between alcohol consumption and mortality in AAAD patients.

Cardiogenic shock was recognized as another predictive factor of early mortality, which is mostly due to pericardial tamponade or a high-grade aortic regurgitation. Several studies addressed the adverse relationship between preoperative shock, pericardial tamponade, and pre-admission ventilation and the survival of AAAD patients^[22]^.

Also, CAD was associated with early death. It can be a result of either concomitant coronary atherosclerosis or a consequence of aortic sinus laceration due to extensive dissection process, which in turn resulted in a longer operating time and more difficult surgery.

Leukocytosis on admission was associated with early mortality in our patients’ population. This might be explained by the inﬂammatory process, which plays a significant role in AD pathogenesis. An elevated white blood cells (WBCs) count has been observed in dissection patients as soon as the onset of the syndrome, suggesting a very early initiation of a cascade of inflammation in AAAD. The tissue destruction and thrombi in the false lumen created by the dissection might induce the inﬂammatory reaction. The activated circulating WBCs adhered to endothelium and damaged it with toxic oxygen compounds and proteolytic enzymes, this contributed a lot to the injury of the tissues. The WBCs, such as neutrophils and macrophages, have been detected in teared aortic tissues. WBCs were also responsible for the extension of the false lumen, which clearly indicated that WBCs may reﬂect the severity in cases of acute AD.

On the other hand, acute AD might cause malperfusion syndrome; significant WBCs elevation has been observed in the end-organ ischemic complications that resulted from cerebral, visceral, or coronary malperfusion. Higher WBCs in patients with type A AD might reﬂect the higher severity of malperfusion syndromes when there is end-organ involvement. Guan et al.^[23]^ demonstrated that high WBC count was a significant predictor of higher rate of postoperative neurological complications. During the surgery, the inflammatory cytokines and cells might add insult to ischemia-reperfusion injury and lead to poor prognosis.

Our study has also demonstrated that elevated WBC count is another independent predictor of early mortality. The high leucocytic count finding and its relationship with early mortality is consistent with results from Fan et al. ^[24]^.

Concerning intraoperative risk factors for death, various studies with similar results to ours also reported that whether the preservation or replacement of the aortic root, ascending aorta, and either the total or hemiarch replacement were not independent predictors of mortality, neither were the application of different methods of cerebral perfusion or the arterial cannulation site^[14,25]^.

While univariate logistic regression analysis showed that longer operation times (whole surgery, CPB time, and aortic cross-clamp time) were highly associated with early mortality, the total operation time remained as an independent predictor of early death in the multivariate logistic regression (*P*<0.008).

Postoperatively, on univariate analysis, respiratory problems in the form of prolonged mechanical ventilation, pulmonary infection, and the need for tracheotomy were also associated with early mortality. Readmission on the intensive care unit was another indicator of consecutive death. Stroke and ARD were highly significant predictors of mortality, both on univariate and multivariate analyses.

### Limitations

We need to acknowledge some limitations of our study. First, the retrospective observational nature is the main limitation of this study. Second, this is a single-center experience on a relatively small sample. Third, the dichotomous nature of data as regards to alcohol drinking habits, which must be investigated in more details.

## CONCLUSION

The use of ESM did not result in any rise in early death or adverse events compared with other surgical approaches in the literature.

Despite the advantages of ESM for AAAD over the other less aggressive surgical approaches, the surgical team should consider the patient’s preoperative comorbidities and other risk factors for early mortality, in particular, prolonged operation time.

A positive relationship was found between preoperative regular alcohol intake and early mortality in these surgical candidates; this is - to the best of our knowledge - the first study to report this relationship.

**Table:** 

Author's roles & responsibilities	
ASA	Substantial contributions to the conception or design of the work; or the acquisition, analysis, or interpretation of data for the work; drafting the work or revising it critically for important intellectual content; final approval of the version to be published
FX	Substantial contributions to the conception or design of the work; final approval of the version to be published
XW	Agreement to be accountable for all aspects of the work in ensuring that questions related to the accuracy or integrity of any part of the work are appropriately investigated and resolved; final approval of the version to be published
